# Ferroptosis Mechanisms Involved in Neurodegenerative Diseases

**DOI:** 10.3390/ijms21228765

**Published:** 2020-11-20

**Authors:** Cadiele Oliana Reichert, Fábio Alessandro de Freitas, Juliana Sampaio-Silva, Leonardo Rokita-Rosa, Priscila de Lima Barros, Debora Levy, Sérgio Paulo Bydlowski

**Affiliations:** 1 Lipids, Oxidation, and Cell Biology Group, Laboratory of Immunology (LIM19), Heart Institute (InCor), Hospital das Clínicas HCFMUSP, Faculdade de Medicina, Universidade de São Paulo, São Paulo 05403-900, Brazil; kadielli@hotmail.com (C.O.R.); fabio.alessandro@usp.br (F.A.d.F.); jukisbio@gmail.com (J.S.-S.); rokita@usp.br (L.R.-R.); pri_limabarros@hotmail.com (P.d.L.B.); d.levy@hc.fm.usp.br (D.L.); 2 Instituto Nacional de Ciencia e Tecnologia em Medicina Regenerativa (INCT-Regenera), CNPq, Rio de Janeiro 21941-902, Brazil

**Keywords:** ferroptosis, cell death, iron metabolism, neurodegenerative diseases, glutathione peroxidase 4, GSH, system xc^−^, Alzheimer’s disease, Parkinson’s disease, Huntington’s disease

## Abstract

Ferroptosis is a type of cell death that was described less than a decade ago. It is caused by the excess of free intracellular iron that leads to lipid (hydro) peroxidation. Iron is essential as a redox metal in several physiological functions. The brain is one of the organs known to be affected by iron homeostatic balance disruption. Since the 1960s, increased concentration of iron in the central nervous system has been associated with oxidative stress, oxidation of proteins and lipids, and cell death. Here, we review the main mechanisms involved in the process of ferroptosis such as lipid peroxidation, glutathione peroxidase 4 enzyme activity, and iron metabolism. Moreover, the association of ferroptosis with the pathophysiology of some neurodegenerative diseases, namely Alzheimer’s, Parkinson’s, and Huntington’s diseases, has also been addressed.

## 1. Introduction

The current classification system of cell death has been updated by the Nomenclature Committee on Cell Death (NCCD), according to their guidelines for the definition and interpretation of all aspects of cell death [1,2]. Accidental cell death (ACD) is an instantaneous and catastrophic demise of cells exposed to severe insults of physical or mechanical forces. On the other hand, regulated cell death (RCD) is a dedicated molecular machinery [3]. RCD can occur in two ways: firstly, as programmed cell death that can occur in the absence of any exogenous environmental perturbation. Secondly, RCD can originate from disturbances of the intracellular or extracellular microenvironment that cannot be restored to cellular homeostasis [4,5,6].

In 2012, Brent R. Stockwell described a unique form of cell death that results from the overwhelming iron-dependent accumulation of lethal amounts of lipid-based reactive oxygen species and named it ferroptosis [7]. Ferroptosis is morphologically and biochemically distinct from other RCDs. It occurs without the chromatin condensation and nuclear reduction seen in apoptosis, cellular and organellar swelling of necrosis, and without the common features of autophagy. Morphologically, only mitochondrial shrinkage distinguishes it from other forms of death [8,9]. Ferroptotic cell death is associated with the iron-dependent mechanism and formation of extremely reactive free radicals, along with severe peroxidation of membrane phospholipids (PLs) rich in polyunsaturated fatty acids (PUFAs), mainly of arachidonic or adrenic acids from phosphatidyl ethanolamine (PE) molecules [10,11,12].

The complex balance between reactive oxygen species (ROS) and the antioxidant system maintains cell homeostasis by removing dangerous stimuli and controlling oxidative stress by several factors, and is also present in the central nervous system (CNS) [13,14]. Among these factors, system xc^−^, an amino acid antiporter, maintains the synthesis of glutathione (GSH) and oxidative protection. Inhibition of system xc^−^ causes a rapid drop of intracellular glutathione levels and cell death caused by the accumulation of lipid-derived ROS. Lipid and protein oxidation lead to inflammation and changes in DNA, and are the trigger for premature aging, loss of function and death of neurons. The increase in oxidative stress generated by free radicals associated with uncontrolled intracellular iron metabolism has been associated with the pathophysiology of neurodegenerative diseases [13,14,15]. Here we address the main pathways of ferroptosis and its role in the pathophysiology of Alzheimer’s disease, Parkinson’s disease, and Huntington’s disease, the main neurodegenerative diseases in which ferroptosis has been shown to be involved.

## 2. Ferroptosis

**Figure 1:**
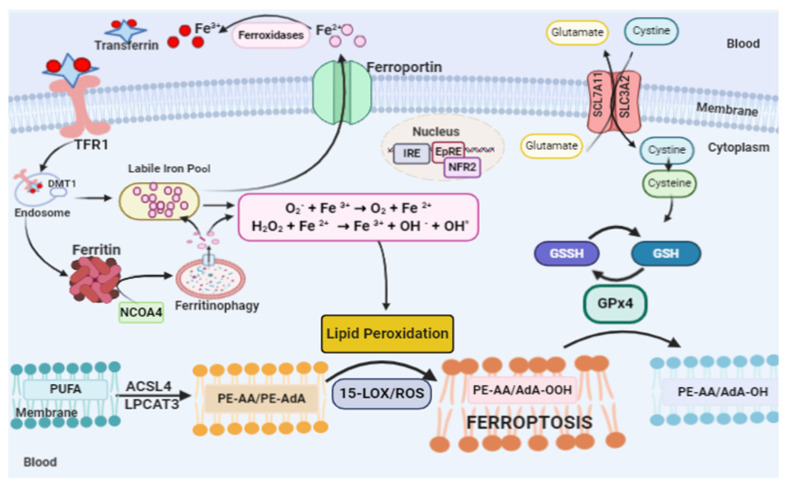
Ferroptosis pathway. Ferroptosis can be initiated through transferrin endocytosis linked to transferrin receptor 1 (TFR1). After endocytosis, ferric iron is released from the Transferrin–TRF1 complex and is reduced to ferrous iron (Fe^2+^). Fe^2+^ can be stored in ferritin or remain in the cytoplasm as a labile iron Pool (LIP). The LIP is composed mainly of Fe^2+^, which through Fenton reaction generates species such as: the hydroxyl radical that reacts with membrane lipids, providing the lipid peroxidation of arachidonic acid (AA) or adrenic acid (AdA). Lipid peroxidation can also occur via enzyme. However, it is necessary for the free polyunsaturated fatty acids (PUFAs) to be esterified as membrane PUFA by the enzymes ACSL4 and LPCAT3, forming arachidonic or adrenic acids esterified in phosphatidyl ethanolamine (PE-AA/PE-AdA). Dioxigenation by 15-LOX generates PE-AA/AdA-OOH, which reacts with other membrane lipids, forming pores in the lipid bilayer, destabilizing it and then rupturing the membrane. Ferroptosis is inhibited by GPx4, which converts PE-AA/AdA-OOH to alcohol and water. This reaction occurs through the use of glutathione (GSH) as a substrate. GSH synthesis occurs via the entry of cystine into the cell by system xc^−^.

Ferroptosis is a particular form of cell death that is induced by lipid hydroperoxides derived from the oxidation of reactive species generated by free iron. Cell death in ferroptosis involves three main factors: increased free intracellular iron, depletion of the redox glutathione/GPx4/system xc^−^ and the oxidation of membrane PUFAs [16,17,18] (Figure 1).

### 2.1. Lipid Peroxidation and Ferroptosis

Lipid peroxidation is the trigger for the activation of ferroptosis [13,18]. Lipid peroxides (PL-OOH), mainly lipid hydroperoxides (L-OOH), have the ability to cause damage to the lipid bilayer of the plasma membrane due to the accelerated oxidation of the membrane lipids which leads to ferroptosis. The increase in the concentration of lipid peroxides can alter the structure and function of nucleic acids and proteins, as well as the Michael acceptors and aldehydes. In fact, it can generate additional toxicity due to its degradation products [19,20,21,22]. The cellular lipids include thousands of lipid species that vary in quantity, intra- and extracellular distribution, functions and cell type [8]. Thus, the higher the concentration of free polyunsaturated fatty acids (PUFAs) in the cell, the greater the damage caused by lipid hydroperoxidation and the extent of ferroptosis, which can vary among diseases and organs/tissues [4,8,17].

PUFAs are good substrates for autoxidation because the C–H bonds of the methylene groups flanked by C-C double bonds are among the weakest C–H bonds known [19,20,21,22]. The structure of the PUFA molecule contains bis-allyl hydrogen atoms that can be abstracted. Then, there is a rearrangement of the resonance radical structure, with subsequent addition of molecular oxygen, giving rise to the peroxyl radical and the formation of the primary molecular product, lipid hydroperoxide (L-OOH). Soon after, the cleavage of the L-OOH molecule occurs, giving rise to highly electrophilic secondary oxidation products, including epoxy, oxo- or aldehyde groups, which are highly reactive and toxic to membranes and cells [8,23].

First, PUFAs are esterified with membrane phospholipids, such as phosphatidyl ethanolamine (PE). The esterification reaction is catalyzed by acyl-CoA synthetase long-chain family member 4 (ACSL4), which binds coenzyme A to long-chain PUFAs, which can then be used for esterification of lysophospholipids by lysophosphatidylcholine acyltransferase 3 (LPCAT3); the substrates can undergo peroxidation resulting in the formation of arachidonoyl (AA) and adrenoyl (AdA) acids, which can lead to ferroptosis. Suppression of the ACSL4 enzyme inhibits ferroptosis by depleting the substrates for lipid peroxidation [24,25].

The PUFA oxidation process that leads to ferroptosis can occur enzymatically or non-enzymatically [26]. The non-enzymatic oxidation process occurs through ROS and hydroxyl radical, from the Fenton reaction. This process is both non-selective and non-specific. Thus, oxidation rates are proportional to the number of readily abstractable bis-allyl hydrogens in the PUFA molecule, resulting in the accumulation of a highly diversified pattern of oxidation products with the predominance of oxygenated PUFA-PLs with 6, 5, 4, 3 and 2 double bonds [8,19]. Enzymatic oxidation of PUFAs occurs through lipoxygenases (LOXs) [27]. LOXs are dioxigenases containing iron in their catalytic region that promote the dioxigenation of polyunsaturated fatty acids containing at least two isolated cis-double bonds. In humans, there are different isoforms of LOX (5-LOX, 12S-LOX, 12R-LOX, 15-LOX-1, 15-LOX-2 and eLOX3) [19,27].

Membrane ester lipids are cleaved by cytosolic phospholipase A2 in different fatty acids: arachidonic acid (AA), eicosapentaenoic acid (EPA) and docosahexaenoic acid (DHA). Oxygenation by cyclooxygenases (COXs) generates prostanglandins-G (PGG2, PGG3 and PGG4, respectively). However, oxygenation by LOX generates doubly and triply oxygenated (15-hydroperoxy)-diacylated PE species [28,29,30,31]. Oxidation induced by 15-LOX is selective and specific, occurring preferably in arachidonic acid-phosphatidylethanolamine (AA-PE) or adrenoyl acid (AdA)-PE. The product of this oxidation is 15-hydroperoxy-arachidonic acid-phosphatidylethanolamines (15-HOO-AA-PEs) or 15-hydroperoxy-adrenoyl acid-phosphatidylethanolamines (15-HOO-AdA-PEs) (Figure 1) [28,29,30,31]. The catalytic activity 15-LOX is dependent on the pro-ferroptotic PEBP1 protein [32].

Stoyanovsky et al. [33] showed that the ferroptosis process includes two stages: (i) selective and specific enzymatic production of 15-HOO-AA-PE by 15-LOX; (ii) oxidative cleavage of these initial HOO derivatives to proximate electrophiles capable of interacting with protein targets to cause the formation of pores in plasma membranes, or to rupture them. The two types of oxidatively truncated products can be formed from 15-HOO-AA-PE with the carbonyl function either on the shortened AA-residue esterified into PE, or on the leaving aldehyde.

In addition, tocopherols and tocotrienols suppress LOX and protect against ferroptosis [24]. On the other hand, ferrostatins inhibit ferroptosis by efficiently scavenging free radicals in lipid bilayers [34].

Recently, Zou et al. [35] have shown that cytochrome P450 oxidoreductase (POR) is a key mediator in the induction of ferroptosis in cells that exhibit intrinsic and induced susceptibility to ferroptosis by enabling membrane polyunsaturated phospholipid peroxidation. POR depletion suppressed arachidonic acid-induced sensitivity to ML210/RSL3 in a dose-dependent manner. In addition to suppressing PUFA-induced ferroptosis susceptibility, POR depletion by constitutive or inducible knockout also compromised the intrinsic ferroptosis sensitivity in ccRCC cells 786-O and 769-P.

### 2.2. Glutathione Peroxidase 4 and Ferroptosis

Cells have several escape mechanisms against cell death [36,37]. In the ferroptotic process, one of the most important and most studied so far is the enzyme glutathione peroxidase 4 (GPx4), (also called Phospholipid Hydroperoxide Glutathione Peroxidase (PHGPx)) [38,39]. In the human organism, there are several isozymes of glutathione peroxidase, which vary in cell location and substrate specificity [40]. The GPx4 enzyme is a selenoprotein, with approximately 20–21 kDa, composed of 197 amino acids, and encoded by the GPx4 gene in chromosome 19 localization [41]. GPx4 has in its active site the amino acid selenocysteine, which is necessary for protection against ferroptosis [42]. The catalytic site of selenocysteine involves three different redox states: selenol, selenenic acid and seleninic acid. These different forms of the redox state allow the regulation of the catalytic efficiency of the peroxide reduction, which is dependent on the cellular redox state [43]. The enzymatic activity of GPx4 is vital to cells, since the enzyme can reduce H_2_O_2_ and is the only enzyme that can reduce phospholipid hydroperoxides [44].

**Figure 2:**
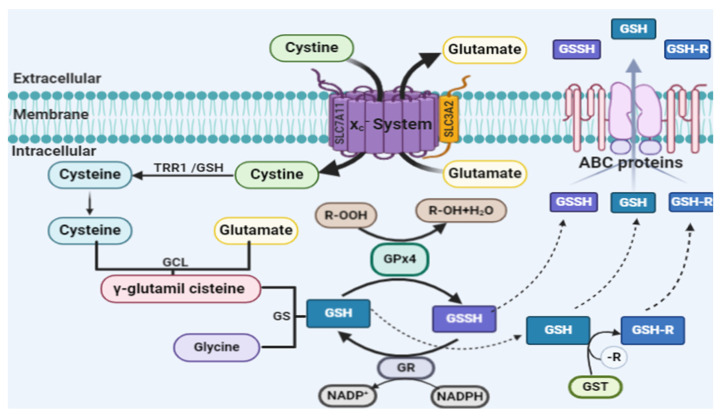
Glutathione (GSH) biosynthesis pathway. GSH is known as one of the small-molecule water-soluble antioxidants, the most important of somatic cells. GSH is a linear tripeptide formed by three amino acids: glutamic acid, cysteine and glycine. The thiol group present in the amino acid cysteine is considered the active site responsible for the antioxidant biochemical properties of glutathione. In biological systems, glutathione can be found in reduced form (GSH) or in oxidized form (GSSG). The oxidized form is a heterodimerization of the reduced form. The GSH/GSSG ratio is used to estimate the redox state of biological systems [51]. The rate-limiting compound of GSH synthesis is the non-essential amino acid cysteine. Cysteine can be imported into cells directly or in its oxidized form, cystine, through the cystine/glutamate antiporter system xc^−^. In humans, on chromosome 4, the SLC7A11 gene (solute carrier family 7A11) encodes the SLCA11 antiporter, which is part of a system called system xc^−^. The structure of this protein is heterodimeric and includes two chains: a specific light chain, xCT (SLCA11), and a heavy chain, 4F2hc (SLC3A2), which are linked by a disulfide bridge. The xCT chain has 12 transmembrane domains consisting of 501 amino acids, with the N and C terminal regions located intracellularly; it is not glycosylated and has a molecular mass of approximately 55 kDa. The heavy chain, 4F2hc, is a type II glycoprotein with a single transmembrane domain, an intracellular NH 2 terminal and a molecular weight of approximately 85 kDa. The 4F2hc chain is a subunit common to amino acid transport systems, while the xCT chain is unique to cystine/glutamate exchange. System xc^−^ transports amino acids, independently of sodium and dependent on chloride, which are specific to import cystine and export glutamate at the same time through the plasma membrane. Both amino acids are transported in anionic form. The ratio of counter transport between cystine and glutamate is 1:1. Currently, it is known that system xc^−^ is involved in (a) cystine uptake to maintain the extracellular balance of cysteine/redox cystine, (b) cysteine/cystine uptake for GSH synthesis and (c) non-vesicular glutamate export [68]. Within the cell, cystine is reduced to cysteine. This reduction reaction can be performed by intracellular GSH or by the enzyme thioredoxin reductase 1 (TRR1) [69]. The beginning of GSH synthesis is the formation of the γ-glutamylcysteine molecule, which is catalyzed by the enzyme glutamate cysteine ligase (GCL). GCL catalyzes the binding of glutamate and cysteine in the presence of adenosine triphosphate (ATP). Then, the enzyme GSH synthase (GS) catalyzes the formation of GSH through the link between γ-glutamylcysteine and glycine [69]. GSH reduces radicals (R•) non-enzymatically and organic hydroperoxides catalyzed by GSH peroxidase (GPx) and is thus converted to GSH disulfide (GSSG). GSSG is recycled to GSH by GSH reductase (GR), a reaction that uses reduced nicotinamide adenine dinucleotide phosphate (NADPH) as a cofactor [69]. GSH S-transferase (GST) forms GSH (GS-R) adducts from organic molecules (R) and GSH, which together with GSH and GSSG are exported from the cell by ABC transporters, mainly ABC-1 and ABC-G2 [70,71]. Extracellular GSH is metabolized by the γ-glutamyl transferase (GGT) ectoenzyme, which transfers the γ-glutamyl residue to different acceptor amino acids, leading to the formation of a dipeptide containing γ-glutamyl and the cysteine glycine dipeptide, which is cleaved by extracellular dipeptides to generate cysteine and glycine that can be taken up by cells, starting the glutathione biosynthesis cycle [69].

In addition, by structural similarity, GPx4 can reduce both peroxidized fatty acids and esterified cholesterol hydroperoxides, as well as thymine hydroperoxide, a product of free radical attack on DNA. The reduction reaction can occur in membranes, in the cytoplasm and/or in lipoproteins [45,46]. In the antiferroptotic process, the GPx4 enzyme directly reduces toxic lipid peroxides (PL-OOH) to non-toxic lipid alcohols (PL-OH) using reduced glutathione (GSH) as a substrate [47,48,49]. The synthesis of GSH through the cystine/glutamate antiporter system xc^−^ is a limiting step for the function of detoxification of lipid peroxides by GPx4 [50]. The rate-limiting compound of GSH synthesis is the non-essential amino acid cysteine. Cysteine can be imported into cells directly or in its oxidized form, cystine, through system xc^−^. Within the cell, cystine is reduced to cysteine by biosynthesis of GSH [51]. Figure 2 shows the complete GSH biosynthesis pathway.

GPx4 inhibitors, including ML210, ML162 and (1S), (3R)-RSL3 (RSL3), are used as specific ferroptosis inducers [52,53,54,55]. Moreover, the overexpression or silencing of the gene coding for 14-3-3 proteins controls the inactivation of GPx4 by RSL3 [56]. In addition, liproxstatin-1 is able to suppress ferroptosis in cells, inhibits mitochondrial lipid peroxidation, and restores the expression of GSH, GPX4 and ferroptosis suppressor protein 1 [57,58]. A variety of ferroptosis inducers can inhibit cystine absorption by inhibiting system xc^−^, such as: erastin, sulfasalazine and sorafenib, resulting in reduced GPx4 activity in different cells lines. Thus, there is no synthesis of GSH and the activity of GPx4 decreases. As a consequence, there is a reduction in the cell antioxidant capacity and hence increased L-ROS, ultimately leading to ferroptosis [59,60,61,62,63,64,65,66,67].

GSH biosynthesis is regulated by the ubiquitously expressed transcription factor nuclear factor erythroid-2 related factor 2 (Nrf2). In baseline conditions, Nrf2-dependent transcription is suppressed due to proteasomal degradation in the cytosol by Keap1 (kelch-like ECH-associated protein 1). However, due to the exposure to a variety of different stimuli, including oxidative stress, the ubiquitination and degradation of Nrf2 are blocked, leading to the stabilization and nuclear accumulation of Nrf2, where it induces dependent electrophilic response element (EpRE) gene expression to restore cellular redox homeostasis [72]. Nrf2 regulates several steps of GSH biosynthesis transcription enzymes, such as catalytic and regulatory subunits of glutamate cysteine ligase (GCL), GSH synthase, GPx2, GSH S-transferases (GSTs) and GSH reductase (GR) as well as the light-chain subunit of the xc- [72,73,74,75]. Nrf2 is also associated with the regulation of antioxidant enzymes, NADPH: quinine oxidoreductase-1 (NQO-1 and NQO-2) and nicotinamide adenine dinucleotide phosphate oxidase 2 (NOX2). In addition, Nrf2 can regulate iron metabolism enzymes [76,77] and proteins associated with multiple drug resistance (ABCG2, MRP3, MRP4, glutathione S-transferase P (GSTP)) [72,78].

Recently, Doll et al. [79] described an in vitro model, a parallel pathway that included FSP1-CoQ10-NADPH, which cooperates with GPx4 and the glutathione system to suppress lipid peroxidation of phospholipids. Ferroptosis suppressor protein 1 (FSP1) provides protection against ferroptosis induced by the deletion of the GPx4 enzyme via RSL3. This effect is mediated by coenzyme Q10 (CoQ10). The reduced form of the enzyme, ubiquinol, captures lipid peroxyl radicals that mediate lipid peroxidation, while FSP1 catalyzes the regeneration of CoQ10 using NADPH as a cofactor. Moreover, the authors described that the antiferroptotic function of FSP1 is independent of cellular glutathione concentration, GPx4 activity, ACSL4 expression and oxidizable fatty acid content [79]. Coenzyme Q10 is an endogenous lipophilic antioxidant produced in the mevalonate pathway, as well as a part of the mitochondrial respiratory chain, and from the metabolism of fatty acid and pyrimidine [80,81]. Indeed, the homologous proteins MDM2 and MDMX, negative regulators of the tumor suppressor p53, promote ferroptosis by regulating lipid peroxidation by altering PPARα activity. MDM2–MDMX complex inhibition increased the levels of both FSP1 proteins and coenzyme Q10 [82].

### 2.3. Iron and Ferroptosis

**Figure 3:**
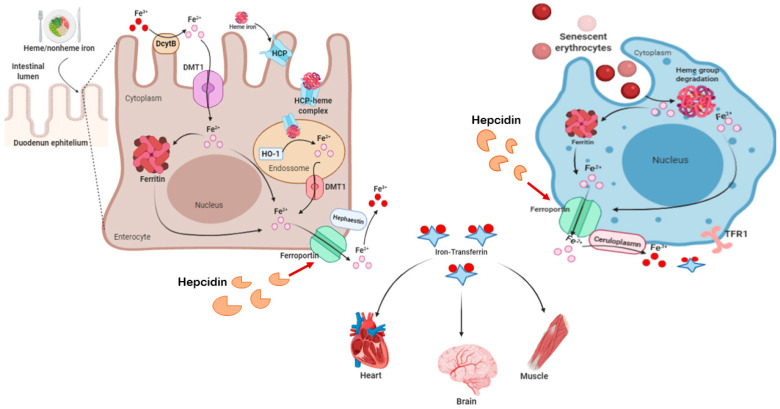
Human Iron metabolism. Iron concentration in the body is maintained through diet and the recycling of senescent erythrocytes. The daily diet provides approximately 1–2 mg of iron. Enterocytes, present in the duodenum and in the proximal portion of the jejunum, can absorb both ferrous iron (heme iron) and ferric iron (non-heme). However, it is necessary to reduce ferric iron to ferrous iron, by apical ferric reductase enzymes, such as enzyme duodenal cytochrome b (Dcytb), for absorption to occur. Then, iron is transported by the divalent metal type transporter-1 (DMT-1) and stored inside the cell [86]. Ferrous iron from the diet is internalized by the heme-1 carrier protein (HCP) in cells, where it is stored as hemosiderin and/or ferritin, to prevent the Fenton reaction. Physiologically, iron stores are mobilized from intracellular to the extracellular by ferroportin (FPN) when the serum iron is low. Iron released in its ferrous state is oxidized to ferric iron and binds to serum apotransferrin to be transported through the body, giving rise to holotransferrin. This oxidation reaction occurs through the action of oxidase enzymes: hephestine present in enterocytes, ceruloplasmin present in hepatocytes macrophages. The distribution of iron to the tissues occurs through the endocytosis of holotransferrin, mediated by the binding to transferrin receptors type 1 (TFR1) and type 2 (TFR2). Ferroportin mediates the efflux of iron within cells, maintaining systemic iron homeostasis. This process is negatively regulated by hepcidin, which promotes ferroportin endocytosis and then proteolysis in lysosomes by induced ubiquitination [83]. The recycling of iron by macrophages occurs through the phagocytosis of senescent erythrocytes and hemoglobin and the heme group of intravascular hemolysis. Once internalized in the macrophage, the heme group releases ferrous iron through the activity of the enzyme heme oxygenase, which can be exported to the extracellular medium by ferroportin or stored as ferritin [83,84].

In the human body, iron metabolism is regulated by means of a perfectly adjusted balance between plasma proteins. They are associated with the transport, absorption and recycling of iron, in order to avoid the accumulation of iron, which is highly harmful and reactive in tissues. Figure 3 shows several aspects of human iron metabolism. Biochemically, iron is capable of accepting and donating electrons, interconverting between the ferric (Fe^3+^) and ferrous (Fe^2+^) forms, which are both found in the human body. The Fe^3+^/Fe^2+^ redox potential participates in a large number of protein complexes, especially those that involve oxygen reduction for adenosine triphosphate (ATP) synthesis and the reduction of DNA precursors. Iron is also a necessary component in the formation of molecules that bind and transport oxygen (hemoglobin and myoglobin) and for the activities of cytochrome enzymes, as well as in many enzymes that perform the redox process, functioning as electron carriers [83,84,85].

Physiologically, iron that will be distributed to tissues needs to bind to transferrin (apotransferrin), giving rise to holotransferrin [87]. The distribution of iron to the tissues occurs through the endocytosis of holotransferrin, mediated by binding to transferrin receptor type 1 (TFR1) and type 2 (TFR2) [88]. After endocytosis, the holotransferrin–TFR1 complex is mobilized to the endosomes. In the acid environment of the endosome, Fe^3+^ is released from TF and converted to Fe^2+^ by oxidation-reduction, by six-transmembrane epithelial antigen of the prostate 3 (STEAP3) and then exported into cytosol by divalent metal transporter 1 (DMT-1). Iron can be stored in ferritin/hemosiderin or remain labile [89].

The labile iron pool (LIP), composed mostly of Fe^2+^, is a pool of chelable iron and active redox present in the cell; it can be present in mitochondria, lysosomes, cytosol and in the nucleus [90]. The concentration of LIP is essentially regulated by the absorption, use, distribution and export of iron in the cell and in the body. Labile iron has high chemical reactivity and exhibits high cytotoxic potential. Cytotoxicity is associated with the fact that labile iron catalyzes the formation of hydroxyl radicals (OH·) derived from hydrogen peroxide (H_2_O_2_) through the Fenton reaction (Figure 1). Moreover, H_2_O_2_ has a lower capacity to react with molecules, while OH· generated from the iron-dependent Fenton reaction has high reactivity with biological molecules, such as proteins and DNA, and generates lipid (hydro) peroxidation in ferroptosis [12,13,14,15,16,17].

Indeed, glutathione has a high affinity with Fe^2+^ and the major component of LIP in cytosol is presented as the glutathione-Fe^2+^ conjugates [90]. This is important because the decrease in intracellular glutathione increases the concentration of Fe^2+^, facilitating the Fenton reaction [90,91,92,93]. The storage of labile iron in ferritin serves to prevent its high reactivity, avoiding the generation of reactive species [94]. The ferritin structure is formed by 24 subunit-composed chains both light (L) and heavy (H) with a spherical “shell” shape, which accommodates about 4500 iron atoms. H-ferritin contains a ferroxidase, which oxidizes Fe^2+^ to Fe^3+^, to store iron inside the nucleus. When necessary, iron stocks are mobilized and exported by ferroportin (FPN). This process is downregulated by hepcidin [83,84,85].

The intracellular iron content is regulated by the iron regulatory proteins (IRP1 and IRP2) and the iron-responsive element (IRE) [95,96]. IRPs can bind to RNA stem-loops containing an IRE in the untranslated region (UTR), affecting the translation of target Mrna: 3′ UTR of H-ferritin mRNA and in the 5′ UTR of TFR1 mRNA. In response to cellular iron demand IRE/IRP interaction promotes TFR1 mRNA stability and inhibits H-ferritin translation, thus modulating cellular iron uptake and storage. Overexpression of both TF and TFR1 sensitizes cells to ferroptosis by enhancing iron uptake; on the other hand, silencing TFR1 can inhibit erastin-induced ferroptosis [16,97,98,99]. In addition, anti-TfR1 antibodies identify tumor ferroptotic cells from different tissues [100].

Autophagy in fibroblasts leads to erastin-induced ferroptosis through the degradation of ferritin and induction of TfR1 expression [101]. On the other hand, cellular senescence has been associated with intracellular iron accumulation and impaired ferritinophagy [102]. Ferritinophagy induces ferroptosis by increasing the nuclear receptor coactivator 4 (NCOA4), followed by an increase in ferritin degradation in the phagolysosome and release of Fe^2+^ (labile iron pool) in the cytoplasm. In fact, some authors consider ferroptosis to be a type of autophagy [103,104,105,106,107].

Another molecule involved in ferroptosis is the sigma-1 receptor (S1R), which protects hepatocellular carcinoma cells against ferroptosis. S1R regulates ROS accumulation via Nrf2. Knockdown of S1R blocks the expression of GPx4 and HO-1. Moreover, knockdown of S1R significantly increases Fe^2+^ levels and MDA production in HCC cells treated with erastin and sorafenib, as well as the upregulation of H-ferritin chain and TRF1 [108]. In addition, it has been shown that heat shock protein β-1 (HSPβ1) is a negative regulator of ferroptotic cancer cell death, and erastin stimulates heat shock factor 1 (HSF1)-dependent HSPβ1. Knockdown of HSF1 and HSPβ1 enhances erastin-induced ferroptosis, whereas heat shock pretreatment and overexpression of HSPβ1 inhibits erastin-induced ferroptosis by protein kinase C. Moreover, the increase in cellular iron in HSPβ1 knockdown cells has been associated with increased expression of TFR1 and a mild decrease in the expression of the H-ferritin chain [109]. HSPA5, an endoplasmic reticulum (ER)-sessile chaperone, was shown to bind and stabilize GPx4, thus indirectly counteracting lipid peroxidation in ferroptosis [97].

## 3. Ferroptosis in Neurodegenerative Diseases

Iron is vital to the physiology of all human tissues. However, under certain conditions it can be harmful, especially for the brain. Although the cellular metabolism of the CNS requires iron as a redox metal for energy generation, mainly the production of ATP, nervous tissue is vulnerable to oxidative damage generated by excess iron and decreased antioxidant systems [110,111,112,113]. The explicit identification of ferroptosis in vivo is hampered by the lack of specific biomarkers, due to several factors that may be associated with the ferroptotic process. However, there is considerable evidence that implicates ferroptosis in the pathophysiology of neurodegeneration. Ferroptosis involves the accumulation of brain iron, glutathione depletion and lipid peroxidation simultaneously, which triggers a cascade of events including activation of inflammation, neurotransmitter oxidation, neuronal communication failure, myelin sheath degeneration, astrocyte dysregulation, dementia and cell death. Iron or free iron overload can initiate lipid peroxidation in neurons, astrocytes, oligodendrocytes, microglia and Schwann cells. In addition, the low activity of GPx4 and the glutathione system have been shown to be associated with ferroptosis in motor neurodegeneration [114,115,116,117].

Recently, it has been proposed that the modulation of ferroptosis may be beneficial for neurodegenerative diseases and that inhibition of ferroptosis by GPx4 could provide protective mechanisms against neurodegeneration [118]. First, it was demonstrated that the non-oxidative form of dopamine is a strong inhibitor of ferroptotic cell death. Dopamine reduced erastin-induced ferrous iron accumulation, glutathione depletion, and malondialdehyde production. Moreover, dopamine increased the stability of GPx4 [119]. The GPx4 enzyme is essential for the survival of parvalbumin-positive interneurons and prevention of seizures, as well as protection against ferroptosis in animal models [42,120]. Next, in a study with PC12 cell line (a model system for neurobiological and neurochemical studies), cell death was induced by *tert*-butylhydroperoxide (t-BHP), a widespread inducer of oxidative stress; it was observed that t-BHP increased the generation of lipid ROS, decreased the expression of GPx4 and the ratio of GSH/GSSG. All these effects could be reversed by the ferroptosis inhibitor, ferrostatin-1 and deferoxamine, iron chelator. In addition, JNK1/2 and ERK1/2 were activated upstream from the ferroptosis and mitochondrial dysfunction [121].

In addition, in C57BL/6 J male mice treated with arsenite for 6 months, it was observed that arsenite induced ferroptotic cell death in neurons by the accumulation of reactive oxygen species and lipid peroxidation products, disruption of Fe^2+^ homeostasis, depletion of glutathione and adenosine triphosphate, inhibition of system xc^−^, activation of mitogen-activated protein kinases and mitochondrial voltage-dependent anion channel pathways, and upregulation of endoplasmic reticulum stress [122]. This is an important issue because arsenite (inorganic arsenic) has been associated with neural loss and Alzheimer’s and Parkinson’s diseases as well as amyotrophic lateral sclerosis (ALS) [123,124,125,126,127,128,129,130]. An in vitro study showed that exposure to paraquat and maneb induced ferroptosis in dopaminergic SHSY5Y cells, associated with the activation of NADPH oxidase. The activation of NADPH oxidase contributed to the dopaminergic neurodegeneration associated with lipid peroxidation and neuroinflammation [131].

In a multiple sclerosis model and in an experimental autoimmune encephalomyelitis (EAE) animal model [124], it has been observed that mRNA expression of the cytoplasmic, mitochondrial and nuclear GPx4 enzyme decreased in multiple sclerosis gray matter and in the spinal cord of EAE. Neuronal GPx4 was lower in EAE spinal cords than in controls. Moreover, γ-glutamylcysteine ligase and cysteine/glutamate antiporter were diminished in EAE, which is associated with high accumulation of lipid peroxidation products and the reduction in the proportion of the docosahexaenoic acid in non-myelin lipids. These results, together with the presence of abnormal neuronal mitochondrial morphology, which includes an irregular matrix, ruptured external membrane and reduced/absent ridges, are consistent with the occurrence of ferroptotic damage in inflammatory demyelinating disorders [132].

In fact, iron can bind to IRPs, leading to the dissociation of IRPs from the IRE and altered translation of the target transcripts. Recently, an IRE was found in the 5′-UTR of the amyloid precursor protein (APP) and α-synuclein transcripts (α-Syn). The levels of α-Syn, APP and amyloid β (Aβ) peptide, as well as protein aggregation, can be negatively regulated by IRPs, but are regulated positively in the presence of iron accumulation. Therefore, it has been suggested that the inhibition of the IRE-modulated expression of APP and α-Syn or iron chelation in patient brains has therapeutic significance for human neurodegenerative diseases [133].

### 3.1. Ferroptosis in Alzheimer’s Disease

**Figure 4:**
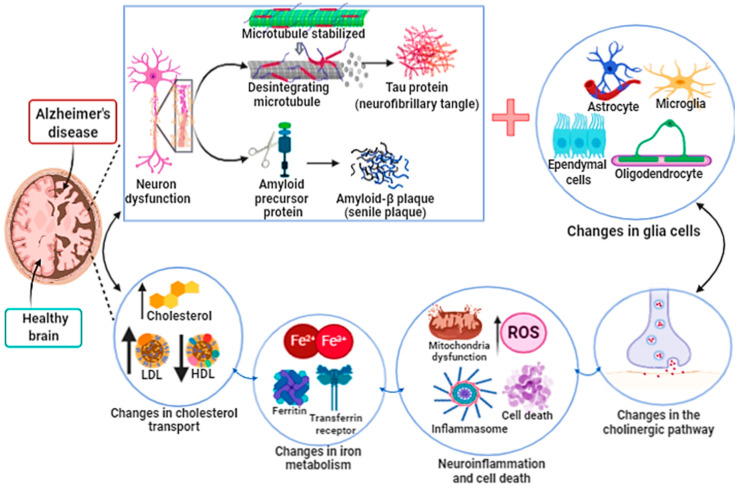
Alzheimer’s disease. The development and progression of Alzheimer’s disease (AD) lead to atrophy, loss and dysfunction of both neurons and glial cells. AD begins in the dorsal raphe nucleus with subsequent progression to the cortex, which is the center of information processing and memory storage. The factors that promote the development of AD are still unknown. However, it seems that the intracellular accumulation in neurons of the phosphorylated Tau protein (neurofibrillary tangle) and the formation of amyloid-B plaque (senile plaque) in the extracellular environment and brain tissue both lead to neuron loss and dysfunction. In addition, the formation of neurofibrillary tangle and senile plaque alters the functions of glial cells, such as oligodendrocytes (responsible for the myelination of neurons), microglia cells (phagocytic cells) and astrocytes (responsible for the absorption and exchange of nutrients between neurons and blood vessels). Dysregulation of cholesterol transport and iron metabolism in the central nervous system contributes to poor prognosis of Alzheimer’s disease. All these associated factors lead to an increase in neuroinflammation and oxidative stress associated with mitochondrial dysfunction, compromising the production of ATP, altering the concentration of neurotransmitters in the synaptic cleft, finally promoting cell death.

Alzheimer’s disease (AD) is considered a neurodegenerative disease associated with multiple brain complications. It was initially described by the German Alois Alzheimer in 1907 [134,135]. AD is characterized by progressive disorder in the cortical and hippocampal neuronal areas which leads to both loss of neuronal function and cell death, and is the most common type of dementia (Figure 4). The hallmark of AD is the histopathological presence of an extracellular β-amyloid (Aβ) deposition in senile plaques (SPs) and intracellular neurofibrillary tangles (NFTs) formed from the hyperphosphorylation of the tau protein. Neurocognitive decline is associated with synapse decrease and neurotransmitter oxidation. These changes are due to the increase in oxidative stress, mainly an increase in ROS and intra- and extracellular hydrogen peroxides [136,137,138]. Moreover, genetic changes include alterations in amyloid precursor protein (APP), apolipoprotein E (APOE), presenilin 1 (PSEN1) and presenilin 2 (PSEN 2) genes [139].

Evidence of the association between the accumulation of iron in the cerebral cortex and the development of Alzheimer’s disease emerged in the early 1960s [140,141]. Since then, several studies have demonstrated the direct association between free iron, oxidative stress, lipid peroxidation and cell death of neurons, usually associated with apoptosis and/or necrosis, due to increased neuroinflammation. The iron dyshomeostasis is associated with ROS production and neurodegeneration in AD [138]. Furthermore, aging and changes in iron metabolism are associated with the development of Aβ plaques and NFTs. Svobodová et al. [142] demonstrated in an APP/PS1 transgenic mice model that free iron and ferritin accumulation follows amyloid plaque formation in the cerebral cortex area. In fact, iron deposition has been involved in the misfolding process of the Aβ plaques and NFTs [143].

Iron is related to the development of Tau protein and, consequently, NFTs. In fact, iron is present through the induction and regulation of tau phosphorylation [143,144]. The association of NFT with neurodegenerative dysfunctions is termed tauopathy [145,146,147]. The oxidation process slows down or excludes the regular action of the Aβ and tau protein [148]. In animal models of tauopathies, increased iron associated with aging and neurodegeneration has been observed [149]. Indeed, animals with tauopathies treated with the iron chelator deferiprone showed a trend toward improved cognitive function associated with the decrease in brain iron levels and sarkosyl-insoluble tau [150].

APP is a type 1 transmembrane protein and its function in heathy individuals appears to be associated with the development of synaptic activity [145]. Proteolytic cleavage of the β-amyloid precursor protein (APP) to form the β-amyloid peptide (Aβ) is related to the pathogenesis of AD because APP mutations that influence this process induce familial AD or decrease the risk of AD [145]. The amyloid cascade hypothesis states that the agglomeration and production of Aβ plaques in the brain would occur, resulting in cell death. Presenilins 1 (PSEN1) and presenilins 2 (PSEN2) precisely cleave the APP and other proteins as they are part of the catalytic protease compounds [151]. Acetylcholinesterase participates in the aggregation of Aβ plaques [152]. Moreover, the Aβ plaques in the presence of free iron participate efficiently in the generation of ROS resulting in increased lipid peroxidation, protein oxidation and DNA damage [153]. Deferiprone derivatives act as acetylcholinesterase inhibitors and in iron chelation [154].

The proteolytic cleavage in APP occurs by enzymatic complexes involving α-secretase or β-secretase and γ-secretase. The proteolytic cleavage in APP by β-secretase produces a neurotoxic 40 to 42 amino acid amyloid [155]. Tsatsanis et al. [156] showed that APP promotes neuronal iron efflux by stabilizing the cell-surface presentation of ferroportin, and that β-cleveage of APP depletes surface ferroportin, leading to intracellular iron retention independently on the generation of Aβ. Furthermore, these findings indicate how β-secretase’s processing of APP might indirectly promote ferroptosis. Iron overload alters the neuronal sAPPα distribution and directly inhibits β-secretase activity [157]. Cortical iron has been strongly associated with the rate of cognitive decline [158]. Iron in the brain increases lipid peroxidation, oxidative stress, and neuroinflammation due to the depletion of neuronal antioxidant systems—mainly the glutathione system [143]. In addition, increased hepcidin expression in APP/PS1 mice astrocytes improves cognitive decline and partially decreases the formation of Aβ plaques in the cortex and hippocampus. Indeed, decreased iron levels in neurons led to a reduction in oxidative stress (induced by iron accumulation), decrease in neuroinflammation and decreased neuronal cell death in the cortex and the hippocampus. [159]. As mentioned before, the hepcidin peptide binds ferroportin, which is followed by cell internalization and further degradation [160].

In order to investigate whether neurons of the cerebral cortex and hippocampus severely affected in patients with AD may be vulnerable to ferroptosis, Hambright et al. [161] have shown in GPx4BIKO mice (a mice model with a conditional deletion in neurons of the forebrain of GPx4) that tamoxifen led to the deletion of GPx4 mainly in neurons of the forebrain. GPx4BIKO mice exhibited significant deficits in spatial learning and memory function, as well as hippocampal neurodegeneration. These results were associated with ferroptosis markers, such as increased lipid peroxidation, ERK activation and neuroinflammation. In addition, GPx4BIKO mice fed a vitamin E-deficient diet had an accelerated rate of hippocampal neurodegeneration and behavioral dysfunction. On the other hand, treatment with Liproxstatin-1, a ferroptosis inhibitor, improved neurodegeneration in these mice. Moreover, in an in vitro model, iron increased nerve cell death in conditions where GSH levels were reduced, by decreasing the activity of glutamate cysteine ligase [162].

The HT22 cell line has high concentrations of extracellular glutamate, which inhibit the glutamate-cystine antiport, leading to the depletion of intracellular GSH and resulting in excessive ROS production. In a study with these cells, Hirata et al. [163] found that an oxindole compound, GIF-0726-r, prevented cell death induced by oxidative stress, including oxytosis induced by glutamate and ferroptosis induced by erastin. Moreover, an excess of extracellular glutamate associated with high levels of extracellular iron cause the overactivation of glutamate receptors. As a consequence, there was an increase in iron uptake in neurons and astrocytes, increasing the production of membrane peroxides. Glutamate-induced neuronal death can be mitigated by iron chelating compounds or free radical scavenging molecules. Ferroptosis is induced by reactive oxygen species in the excitotoxicity of glutamate [110,164,165]. In addition, the sterubin compound maintained GSH levels in HT22 cell lines treated with erastin and RSL3, suggesting protection against ferroptosis [166]. 7-*O*-esters of taxifolin 1 and 2 were described as having neuroprotective action against ferroptosis induced by RSL3 in HT22 cells [167].

Chalcones 14a–c were shown to inhibit β-amyloid aggregation, and in addition, protect neural cells against toxicity induced by Aβ aggregation and from erastin and RSL3-induced ferroptosis in human neuroblastoma SH-SY5Y cells [160]. The authors suggested that the inhibition of toxicity induced by Aβ plaques’ aggregation and of ferroptosis occurs due to the presence of hydroxyl groups in the chalcone derivatives. Chalcone 14a-c can react with lipid peroxyl radicals by transferring the hydrogen (H) atom, thus inhibiting lipid peroxidation [168].

After treatment with high dietary iron (HDI), WT (wild type) mice and the APP/PS1 double Tg mouse model of ADon (HDI) showed upregulation of divalent metal transporter 1 (DMT1) and ferroportin expression, and downregulation of TFR1 expression, with fewer NeuN-positive neurons in both animal models. Moreover, the iron-induced neuron loss may involve increased ROS production and oxidative mitochondria dysfunction, decreased DNA repair, and exacerbated apoptosis and autophagy [169]. Using X-ray spectromicroscopy and electron microscopy it was found that the coaggregation of Aβ and ferritin resulted in the conversion of the ferritin inert ferric core into more reactive low oxidation states [170].

Ates et al. [171] showed in an animal model that inhibition of fatty acid synthase (FASN) by CMS121 decreased lipid peroxidation. CMS121 treatment reduced the levels of 15LOX2 in the hippocampus compared to those of untreated WT mice. Relative levels of endocannabinoids, fatty acids, and PUFAs were significantly higher in untreated AD mice as compared to CMS121-treated AD mice, suggesting that other enzymes may be involved in the process of ferroptosis in Alzheimer’s disease.

It is important to highlight the heterogeneity of Alzheimer’s disease and the involvement of multiple metabolic pathways which contribute to the poor prognosis of this disease (Figure 4). In fact, multiple patterns of cell death are involved in the neurodegeneration process, such as apoptosis, necrosis, and autophagy associated with disturbed BBB (brain blood barrier) permeability. In vitro experiments are the main evidence of ferroptosis in human neurodegenerative processes. The identification of ferroptosis in in vivo models of Alzheimer’s disease is difficult since specific markers for ferroptotic cells, such as specific antibodies, are not available. In addition, other metal ions, such as copper, can also regulate ferroptosis and lipid peroxidation [172,173]. Taking all under consideration, we still do not know whether ferroptosis is the cause or consequence of neurodegeneration processes such as Alzheimer’s disease.

### 3.2. Ferroptosis in Parkinson’s Disease

Parkinson’s disease (PD) is one of the most common and best-known diseases of the nervous system, affecting roughly 0.1–0.2% of the general population and 1% of the population above 60 years [174]. PD is characterized as a slowly progressing neurodegenerative ailment with motor and non-motor clinical manifestations, due to an intense decrease in dopamine production [175]. Classic hallmarks in PD are still related to the motor manifestation such as bradykinesia, resting tremor and rigidity [176]. However, non-motor symptoms associated with PD have recently gained more attention due to their relevance and impact on the patient’s quality of life. Non-motor symptoms of PD include anosmia, constipation, pain, anxiety, depression, psychosis and cognitive disorders that can progress to dementia [177,178,179].

**Figure 5:**
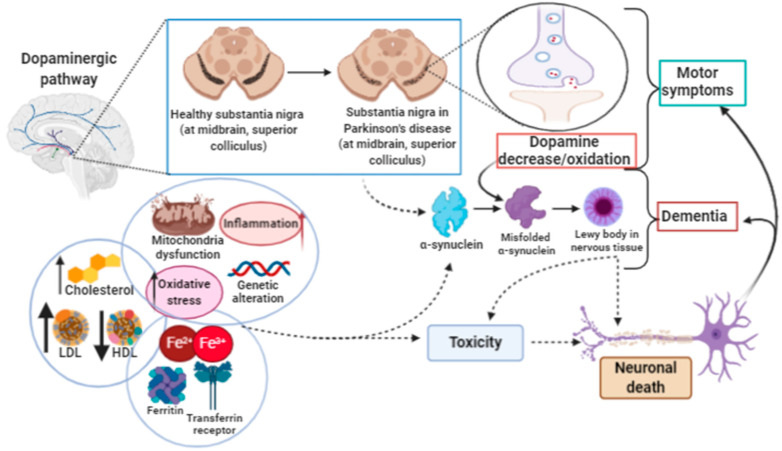
Parkinson’s disease. Parkinson’s disease (PD) occurs due to the decrease in and/or oxidation of dopamine in the substantia nigra, involving the motor system. The incorrect folding of α-synuclein leads to the accumulation of protein (Lewy body) in nervous tissue. The formation of Lewy bodies may be due to a highly pro-oxidative environment, due to dysfunction in the transport of lipids, iron, inflammation and mitochondrial changes. The increase in Lewy bodies is the trigger for the development of dementia, neurotoxicity and neuronal death.

The pathophysiological characteristics of PD include the slow and progressive degeneration of dopaminergic neurons in the pars compacta of the substantia nigra (SNpc), which is associated with a systematic and progressive accumulation of iron, leading to striatum dopamine depletion, disappearance of neuromelanin and the appearance of intracellular Lewy bodies having aggregated α-synuclein as the main component [180,181]. During PD progression there is an increase in oxidative stress, lipid peroxidation, and mitochondrial dysfunction associated with the depletion of antioxidant enzymes in the glutathione systems. All of these associated factors lead to neuronal death and the functional disability of the organism (Figure 5). Currently, the pharmacological treatment of PD aims to increase dopamine levels in the synaptic cleft. Levodopa is the drug of choice, being associated with dopamine agonists, dopamine metabolism inhibitors and decarboxylase inhibitors. Treatment is stable for a period of 5–6 years. Then, however, the disease progresses with marked neurodegeneration and development of dementia [182,183,184].

The association between iron and PD is long-standing [185,186]. Daily exposure to elevated iron levels is a risk factor for the development of PD [187]. In addition, an increase in the iron content in substantia nigra and globus pallidus of PD patients was observed by magnetic resonance imaging (MRI). This increase was associated with time of disease, neurodegeneration and severity of motor impairment [188,189]. In mice, treatment with deferiprone (DFP) was shown to significantly reduce labile iron and biological damage in oxidation-stressed cells, improving motor functions while raising striatal dopamine levels. Furthermore, in patients with PD, a decrease in iron overload has been described in the substantia nigra after 6 months of deferiprone treatment, as has an increase in ceruloplasmin activity in cerebrospinal fluid (CSF) [190,191,192,193].

Ferroptosis is also proving to be a mechanism of immeasurable importance for the pathogenesis of PD [194]. In fact, since the early 2000s it has been known that elevated levels of iron could be found in the brain of patients with PD, although an iron-dependent cell death mechanism had not yet been proposed at that time. Additionally, several genes and proteins related to iron metabolism of brain cells have been found to be mutated in PD patients, strengthening the correlation between iron metabolism and Parkinson’s disease [195,196].

Some previous works on Parkinson’s disease described the presence of PUFA peroxidation, a decrease in GPx4 activity and exhaustion of the glutathione system, associated with increased oxidative stress. The first evidence of ferroptosis in Parkinson’s disease was described by Do Van et al. [197]. PD models, both in vitro and in vivo, have shown that the characteristic features of ferroptosis were present in differentiated Lund human mesencephalic (LUHMES) cells intoxicated with erastin. The characteristics of ferroptosis in LUHMES cells were different from those reported for other cell lines. Moreover, the calcium chelator 1.2-bis(o-aminophenoxy)ethane-N,N,N′,N′-tetraacetic acid (BAPTA) and protein kinase C (PKC) inhibitors (the bisindolylmaleimide analog Bis-III, and small interfering RNA (siRNA)) were very effective in counteracting erastin-induced cell death. In LUHMES cells, ferroptosis requires activated mitogen-activated protein kinase (MEK) signaling but is independent of Ras activation. Moreover, ferroptosis involvement in dopaminergic cell death was observed in a mouse model in which toxicity was inhibited by the specific ferroptosis inhibitor ferrostatin 1. Lastly, the regulation of dopaminergic cell death by ferroptosis and its inhibition by PKC were also confirmed ex vivo by studying organotypic slice cultures (OSCs). It is important to note that the ferroptosis activation pathways were initially described in cancer models, in which there was elevated metabolic activity and cellular proliferation due to uncontrolled cellular repair pathways. These pathways are not activated in neurodegeneration models. Therefore, ferroptosis can be triggered by different mechanisms in different cells and different tissues.

A plethora of new evidence is clarifying the molecular mechanisms involved in the interaction of PD and ferroptosis cell death. α-Synuclein, a protein abundantly expressed in the nervous system and a main component of Lewy bodies, has been widely studied in PD as its pathogenic effects are strongly correlated with PD’s pathophysiology [198]. Additionally, it has been recently shown that α-synuclein aggregation (a common feature in PD) is responsible for the production of ROS followed by lipid peroxidation in an iron-dependent manner, resulting in increased calcium influx and consequent cell death [199]. In this way, the use of ferroptosis inhibitors such as ferrostatin or iron chelators [200] has been sufficient to suppress cell death, supporting the hypothesis that ferroptosis is a major player in this process and may harbor therapeutic potential. Several studies suggest the modulation in ferroptosis as a therapeutic target in neurodegenerative diseases [201,202].

Another molecule that may link PD to ferroptosis is the transcription factor Nrf2. As Nrf2 is involved in the regulation of processes such as the metabolism of iron, lipids and glutathione, several works have focused on understanding how the modulation of this transcription factor can intervene in the ferroptosis pathway [203]. For instance, it was shown that Nrf2 overexpression in brain tumor cells was an indication of poor survival outcomes since Nrf2 bestowed these tumor cells with resistance to cell death mechanisms such as ferroptosis [204]. In addition, the activation of the Nrf2 pathway (p62-Keap1-Nrf2) has also been shown to prevent 6-hydroxydopamine (6-OHDA)-induced ferroptosis in a human dopaminergic cell line (SH-SY5Y) [205]. Taken together, these results demonstrate that the modulation of Nrf2 expression can provide new therapeutic approaches for neurodegenerative diseases such as PD [206]. In addition, studies performed on a monkey model of PD has shown that clioquinol (CQ), a drug primarily used as an antiparasitic agent that also presents iron chelation properties [207], was able to improve both, motor and non-motor manifestations in treated monkeys. Shi et al. [208] observed that CQ not only led to a decrease in iron levels in the substantia nigra but also managed to suppress the well-known oxidative stress present in PD. At a molecular level, CQ was shown to be able to reduce p53-mediated cell apoptosis and to diminish the iron content and oxidative stress by activating the AKT/mTOR pathway, which was found downregulated in the PD monkey model [208]. These results, taken together, point out once again to the involvement of ferroptosis in PD and demonstrate how pharmacological interventions could be useful to revert this outcome. Unfortunately, available therapies for PD patients are only capable of mitigating their symptoms and cannot reverse the loss of dopaminergic cells [209]. Therefore, seeking new therapeutic options that may intervene in this primary process of cell death could potentially change PD treatment.

### 3.3. Ferroptosis in Huntington’s Disease

Huntington’s disease (HD) is an autosomal dominant late-onset neurodegenerative disorder (age of onset: 30–50 years). HD is caused by a polymorphic sequence of three CAG nucleotides in exon 1 of the IT15 gene (Huntingtin (HTT)), which is located at 4p16.3. HD was described by George Huntington in 1872, after he observed in Long Island a rare disease present in some families in the region. He called this disease “hereditary chorea” (Huntington, 1872). The main observed clinical signs are motor disorders (such as involuntary movements), cognitive, emotional, and psychiatric disorders (such as personality change and dementia). Carriers of this disease may also have dysphagia, which leads to weight loss. In patients affected with juvenile HD under the age of 20, the most observed disorders are behavioral disorders, learning difficulties, and often seizures [210,211,212,213,214].

The mutation occurs in exon 1 of the HTT gene; this region is polymorphic and encodes a polyglutamine segment (polyQ)—in this fragment, expansion and generation of mutant proteins that can lead to the development of HD may occur [215]. The huntingtin protein has a molecular weight of ~348kDa, and the expression level is different according to the cell types in which it is found: neuronal cell bodies, dendritic cells, and axons. Inside the cell, the huntingtin protein is located in the cytoplasm and partially in the nucleus and can move between these compartments [216,217,218].

**Figure 6:**
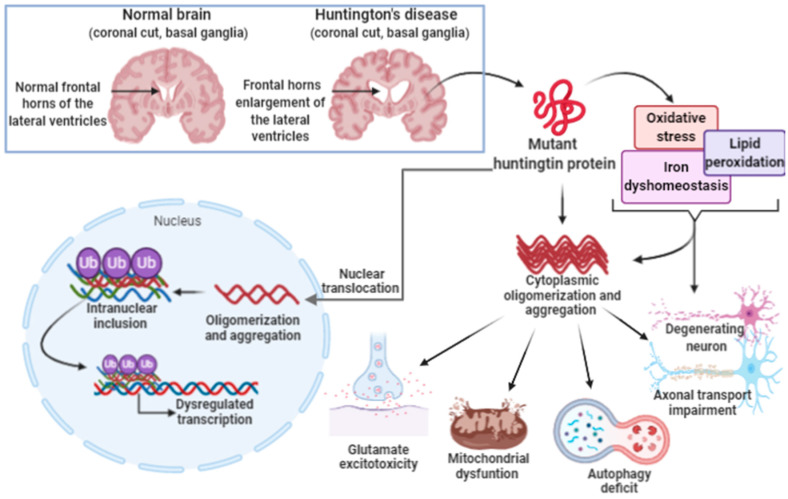
Huntington’s disease. Huntington’s disease is caused by the repetition of autosomal dominant CAG trinucleotide in the Huntingtin gene (HTT gene) on chromosome 4, giving rise to the mutant huntingtin protein. The mutated protein translocates to the nucleus and remains in the cytoplasm. In the nucleus, association, oligomerization and aggregation with other proteins occurs, leading to the formation of inclusions. Protein inclusions disrupt the transcriptional process in nerve tissue cells. In the cytoplasm, the oligomerization, aggregation and precipitation of the huntingtin protein occurs. This process alters the metabolism and both intra- and extra-cellular signaling pathways. The increase in oxidative stress, lipid peroxidation and iron dyshomeostasis contribute to the aggregation and oligomerization of huntingtin protein with other cytoplasmic proteins. Aberrant protein aggregation increases the excitotoxicity of glutamate. Mitochondrial dysfunction changes autophagy mechanisms, and transport in the neuronal axon, leading to nerve cell degeneration.

Such evidence of expression and location suggests that this protein plays an important role in the nervous system, suggesting that changes in its conformation can lead to an imbalance in the performance of its functions, which in turn can result in the development of HD. The pathophysiological mechanism associated with HD includes loss of glial cells (astrocytes and oligodendrocytes), neuronal death, and atrophy of brain tissues, which may start in the striatum, followed by the cerebral cortex (Figure 6). Although mutated huntingtin protein (mHTT) is found in the brain, its expression has been evidenced in cells other than the ones in the central nervous system [216].

One of the cellular processes involved in the development of HD is ferroptosis. A study in mouse models with HD showed an accumulation of toxic iron in neurons compared to the wild model, which suggests that iron accumulation may contribute to the neurodegenerative process [219]. Another study with mice that had GPx4 ablation showed the importance of inhibiting ferroptosis to prevent spinal motor neuron degeneration since mice with this characteristic showed motor disorders. The tests carried out to confirm ferroptosis included the absence of apoptotic markers (caspase-3 and TUNEL) and activation of the ERK pathway [220]. Magnetic resonance images showed an accumulation of iron in the brain of patients with HD [221]. However, the pathway that induces ferroptosis in the brain has not been fully elucidated [222]. The mutated huntingtin protein (mHTT) is cleaved at different points since it has different cleavage sites than those present in the normal protein, which result in fragments with different sizes inside neurons (small oligomers or monomers) [223]. It has been shown that mHTT and the wild version are associated with the outer mitochondrial membrane [224].

Inside the cells, there must be a balance between the state of mitochondrial fission and fusion for the proper functioning of this organelle [225]. When an imbalance occurs, cellular respiration is affected and, consequently, there is an increase in ROS inside cells [225]. Mitochondria can generate significant amounts of ROS, resulting from normal organelle metabolism and the electron transport chain that contributes to oxidative stress. Additionally, an mHTT leads to increased oxidative stress, which consequently increases ROS levels in the cell [226]. Under normal conditions, GSH regulates the activity of GPx4, and its function is to inhibit ferroptosis and eliminate the excess of lipid peroxides [225]. However, an increase in ROS levels and, consequently, an increase in lipid peroxides, which leads to a depletion of GSH, decreases GPx4 [225]. There is a series of intracellular signals that culminate in an imbalance in cell homeostasis and leads to the process of ferroptosis. In patients with HD, there is a deregulation of GSH which interferes with their functions and enzymes dependent on its action [117,225].

In patients with HD and asymptomatic carriers, high lipid peroxidation and low GSH plasma levels have been found, showing that oxidative stress may be linked to the pathophysiological mechanism of HD [227]. Iron chelators could be an alternative for treatment [228].

Nfr2 is a transcriptional regulator of genes involved with ferroptosis, such as GPx4, which can be found in cytosol and modulates mitochondrial function [206]. These are essential components for the process of ferroptosis and demonstrate the importance of Nfr2 in protecting against ferroptosis. Therefore, Nfr2 can be an alternative therapy for reducing ferroptosis [206]. Currently there is no specific treatment available for HD, only palliative care [212]. In general, the multidisciplinary treatment available at the moment is focused on the palliative treatment of symptoms and neuroprotection of patients [218,229]. Although there is no specific treatment for HD, several studies are being developed focusing on silencing the DNA or mRNA of the mutated allele whose gene varies in the number of copies [215,229]. Another promising alternative is the creation of mouse models with HD and the involvement of GSH activity as sources of possible target treatments [117].

## 4. Conclusions and Perspectives

The description of the ferroptosis mode of cell death is still recent. Although its participation in several diseases such as cancer has been increasingly established, in neurodegenerative diseases information is still lacking. Most data have been obtained from experimental studies and more clinical approaches are necessary. For instance, the experimental use of iron chelators and antioxidants has shown to be effective as a possible alternative to interrupt the process of ferroptosis in neurodegenerative diseases. However, when tested in humans, they showed reduced efficacy, which shows that research involving signaling molecules from other pathways that trigger the process of ferroptosis are needed. Nevertheless, there is a growing body of evidence that ferroptosis plays a central role in several neurodegenerative diseases, at least in those described here. This field of research may still yield promising results and disruptive therapeutic alternatives for patients with neurodegenerative diseases.
